# Immunoadsorption study Mainz in adults with post-COVID syndrome (IAMPOCO)—a single-blinded sham-controlled crossover trial to evaluate the effect of immunoadsorption on post-COVID syndrome

**DOI:** 10.1186/s13063-025-08825-7

**Published:** 2025-04-03

**Authors:** Marco Stortz, Pascal Klimpke, Andreas Kommer, Philipp Gründer, Livia Steenken, Christian Dresel, Daniel Kraus, Irene Schmidtmann, Arndt Weinmann, Julia Weinmann-Menke

**Affiliations:** 1 Department for Nephrology, Rheumatology and Kidney Transplantation, I. Unit for Internal Medicine, University Medical Center of the Johannes Gutenberg-University Mainz, Langenbeckstr. 1, Mainz, Germany; 2 Department for Neurology, University Medical Centerof the, Johannes Gutenberg-University Mainzaq , Langenbeckstr. 1, Mainz, Germany; 3 Department for Gastroenterology and Hepatology, I. Unit for Internal Medicine, University Medical Centerof the, Johannes Gutenberg-University Mainzaq , Langenbeckstr. 1, Mainz, Germany; 4 Institute of Medical Biostatistics, Epidemiology and Informatics, University Medical Centre of the Johannes Gutenberg-University Mainz, Langenbeckstr. 1, Mainz, Germany

**Keywords:** Randomized controlled trial, Immunoadsorption, Post-COVID syndrome, Fatigue

## Abstract

**Background:**

Post-COVID syndrome (PCS) affects up to 43% of all SARS-CoV-2-infected persons and describes ongoing symptoms months after the acute infection. Despite the large number of affected people, there is still very little evidence about therapeutic options. Some studies suggest at least partially a role of autoantibody-mediated autoimmunity. Immunoadsorption is an extracorporeal therapy to remove circulating antibodies which is used successfully in several autoimmune diseases. We conceived the IAMPOCO trial to evaluate the therapeutic effect of immunoadsorption in patients with PCS.

**Methods:**

IAMPOCO is a single-center randomized sham-controlled trial with a crossover design which will enroll 40 participants with PCS and a symptom severity of at least 2 on post-COVID functional scale. All participants will undergo 5 immunoadsorption treatments and after a washout period of 8 weeks 5 sham treatments or vice versa. Which modality is conducted first will be randomized. Patients but not providers of therapy are blinded for which modality is conducted. Primary outcome is the efficacy of IA to the severity of PCS measured by the change of several symptom scores and hand grip strength. Secondary outcomes are the frequency of adverse events and the prevalence of relevant autoantibodies in participants with PCS as well as the concentration of autoantibodies before and after therapy and sham treatment.

**Discussion:**

The trial addresses the lack of evidence for treatment options in PCS. By using a crossover design and including a sham treatment arm, the study aims to compare the effects of immunoadsorption and sham therapy within the same patients. The trial also benefits from recruiting participants from a cohort study on PCS prevalence, ensuring a thorough evaluation of symptoms. Objective assessments of symptoms are challenging due to their subjective nature, but various scoring systems and tests are being utilized. Despite the lack of data from RCTs, the results of this study have the potential to significantly improve PCS therapy and support evidence-based treatment decisions.

**Trial registration:**

ClinicalTrials.gov NCT05841498. Registered on May 3, 2023.

**Supplementary Information:**

The online version contains supplementary material available at 10.1186/s13063-025-08825-7.

## Introduction

### Background and rationale {6a}

Post-corona virus disease syndrome (PCS) refers to symptoms that develop 3 months from the onset of corona virus disease-19 (COVID-19) with symptoms that last for at least 2 months and cannot be explained by an alternative diagnosis [1]. The prevalence of PCS is estimated to be 10% of all severe acute respiratory syndrome corona virus-2 (SARS-CoV-2)-infected patients, with hospitalized patients more likely to suffer from persistent symptoms (50–70%) than non-hospitalized patients (10–30%) [2]. Women are more likely to experience PCS than men (incidence, 49% vs 32%, respectively) [2]. With more than 650 million infected people worldwide, PCS is a growing threat to healthcare systems [2, 3]. The most common symptom is fatigue, which affects up to 50% of PCS patients [2]. Other symptoms are memory impairment, dyspnea, sleep disturbances, and joint pain [4]. Headaches, myalgia, anxiety, or depression are also frequently reported [4]. Many patients are limited in their daily lives by these symptoms of PCS and suffer from a diminished quality of life.

While in some patients the etiology of symptoms of PCS appears relatively clear, for example, persistent shortness of breath after a stay in the intensive care unit with weeks of ventilation therapy, other patients complain of persistent symptoms or newly onset symptoms following infection even after very mild courses of the disease. In terms of the type, variety, and duration of symptoms, in many cases PCS resembles a clinical picture observed after various viral infections, such as Epstein-Barr virus, herpes simplex virus, or influenza virus, namely myalgic encephalomyelitis and chronic fatigue syndrome (ME/CFS) [5, 6]. Here, too, patients mainly suffer from fatigue, impaired concentration and memory, and non-restorative sleep [5, 6]. Some authors consider some types of PCS as a form of ME/CFS triggered by the SARS-CoV-2 infection or the immune response to the infection [6]. Similar to ME/CFS, the underlying pathomechanisms of PCS are not fully understood. Autoimmunity is suspected to play a major role in all post-virus syndromes. It may be triggered by the defense against infections and is probably maintained by similarity of endogenous proteins with pathogen components (molecular mimicry) [7]. In the context of this autoimmunity, antibodies against endogenous structures can also be formed, such as antinuclear antibodies, which are directed against components of the cell nuclei [7, 8]. Antibodies against α- and β-adrenergic receptors and muscarinic acetylcholine receptors, among others, have been detected in patients suffering from ME/CFS as well as in patients with PCS [9]. There is growing evidence for at least an involvement of autoantibodies into processes that lead to a specific fatigue-predominant cluster of symptoms of PCS [8, 10].

In several autoantibody-mediated autoimmune diseases like systemic lupus erythematosus, multiple sclerosis, or autoimmune encephalitis, the therapeutic efficacy of immunoadsorption (IA) has been shown [11, 12]. IA is a therapeutic extracorporal procedure which efficiently removes (auto-)antibodies from the circulation by specific binding onto the surface of an adsorber [13]. A few case series have investigated the application of IA to treat symptoms of ME/CFS or PCS but not in a setting with a control-group or crossover design [14–16]. Despite missing evidence, IA is already used, and paid for by patients, to treat PCS in some cases [17]. Whether the treatment of PCS symptoms, especially ME/CFS-like, with IA is effective at all, and which patients might benefit is still completely uncertain. It is important to investigate the therapeutic benefit of this method of therapy in order to make informed treatment decisions and to avoid unnecessary distress to affected individuals in the absence of benefits. Conversely, if benefit is proven, it seems necessary to identify markers or symptom clusters that can indicate which patients will benefit from IA.

### Objective {7}

The objective of the IAMPOCO trial is to investigate the therapeutic effect of IA on symptom severity in the setting of PCS, as well as the prevalence of various autoantibodies in patients with PCS, their association with symptom severity, and whether improvement in symptoms is related to removal of autoantibodies.

### Trial design {8}

The IAMPOCO trial is designed as a prospective single-blinded, randomized sham procedure-controlled trial with a crossover design that evaluates the effect of IA compared to sham procedure on the severity of symptoms of a PCS to investigate whether immunoadsorption is superior to the sham treatment. Persons with PCS are screened for eligibility criteria and subsequently randomized to receive either an initial cycle of immunoadsorption intervention or a sham procedure intervention. After the first treatment cycle, an 8-week washout period is implemented, followed by the alternate intervention. Participants are blinded to the specific intervention they receive.

## Methods: participants, interventions, and outcomes

### Study setting {9}

The study has been conducted from May 2023 to October 2024 at the Medical Center of the Johannes Gutenberg-University Mainz, Germany. The data has been collected and will be analyzed at the same site.

### Eligibility criteria {10}

The inclusion and exclusion criteria for participants are listed in Table 1. For enrolment, all inclusion criteria and none of the exclusion criteria must be met. All IA procedures and sham procedures will be performed by experienced nursing staff under supervision of an experienced physician in the application of extracorporeal blood purification procedures.

**Table 1:** Inclusion and exclusion criteria for participants of the IAMPOCO trial

Inclusion criteria	Exclusion criteria
- Meeting the WHO diagnostic criteria for PCS - Written informed consent to participate in the study - Previous participation in the Gutenberg Post-COVID Study or previously conducted comparable preliminary examinations - Minimum age of 18 years - Value on the post-COVID functional scale of at least 2 (participant is suffering from limitations in everyday life, occasionally need to avoid or reduce usual activities or need to spread these over time due to symptoms, pain, depression, or anxiety; participant is able to perform all activities without assistance)	- Psychiatric diagnosis - Allergy to adsorber materials, materials of tubing systems, or to the substances used for immune adsorption - Pregnancy and breastfeeding - Medical contraindications against immune adsorption such as severe coagulation disorders, intake of angiotensin converting enzyme (ACE) inhibitors, or immunodeficiency syndromes - Preexisting antibody-mediated autoimmune diseases

### Who will take informed consent? {26a}

The study investigators will obtain written informed consent from potential trial participants using the latest version of the approved consent form.

### Additional consent provisions for collection and use of participant data and biological specimens {26b}

N/a. Data and biological samples will be collected and analyzed solely within the scope of the present study. No further analysis beyond this study is intended.

## Interventions

### Explanation for the choice of comparators {6b}

In the present study, IA is the experimental intervention and an extracorporeal sham procedure with the same equipment but without an adsorber is the control intervention. IA has been successfully used to achieve rapid removal of autoantibodies in some antibody-mediated autoimmune diseases [11, 12]. Some studies provide evidence that certain symptoms of PCS may also be antibody mediated [9, 10]. To exclude the possibility that changes in symptom severity are solely due to study participation, examinations, and medical care, a sham adsorption was chosen as the control intervention. In this control intervention, blood is also passed through a machine, exactly the same used for IA without any alteration, to ensure that it cannot be distinguished from actual IA.

### Intervention description {11a}

IA will be conducted with the Plasauto Sigma extracorporeal therapy system in combination with the TR-350 adsorber over 7 days. The therapy is initially conducted on three consecutive days and then twice more on alternate days. During each session, 2–2.5 L of the participant’s plasma will be treated. This regimen has been established by studies with autoimmune diseases [11]. Anticoagulation will be performed with citrate and heparin (1000 iU/h unfractionated heparin with a stopping time of 60 min).

The study has a crossover design. Each participant will undergo a treatment cycle with five IAs and a treatment cycle with five sham treatments with a washout period of 8 weeks in between.

The sham procedure will also be conducted with the Plasauto Sigma extracorporeal therapy system, but without an adsorber in place. To ensure that the sham therapy is indistinguishable from IA for the subjects, the setup is identical. For both IA and sham procedure, the devices are placed behind a portable wall and covered with a curtain so that they are not visible to the patient. However, since the setting-up of the machines differs depending on the procedure, it is not possible to blind the supervising nurse as well.

IA as well as sham procedure will be performed via canulization of peripheral veins using cannulas with a diameter of at least 17 gauge. If this is not possible because of impaired venous conditions, a central venous catheter will be placed in one of the internal jugular veins. Depending on whether a central venous catheter is necessary, the treatments will be performed either inpatient or outpatient.

### Criteria for discontinuing or modifying allocated interventions {11b}

Criteria for discontinuing or modifying interventions are as follows:

1. Participants withdraw their consent.
2. Laboratory findings necessitate an adjustment of the treatment regimen, such as a decrease in fibrinogen concentration, a drop in thrombocyte count, or the occurrence of hypocalcaemia.
3. Worsening of fatigue symptoms, which makes an intervention impossible, especially if it is impossible to travel to the study center.
4. Allergic reactions to any material used for interventions.

### Strategies to improve adherence to interventions {11c}

Strategies to improve the adherence to interventions are as follows:

1. For all participants, detailed information will be provided about the temporal sequence, the purpose, and the benefits of the study. In doing so, the importance of the participation and the adherence for gaining scientific knowledge will be explained.
2. The study participation appointments will be adjusted to the participants’ preferences whenever possible. For employed participants, a sick leave certificate can be provided to accommodate attendance at study-related appointments.
3. Each participant will receive a written overview of the individual schedule of all examinations and interventions required during their study participation at the time of study enrollment. This allows participants to better plan the time required for study participation.

### Relevant concomitant care permitted or prohibited during the trial {11d}

There is no relevant concomitant care which will be provided during the trial. The intake of ACE inhibitors is prohibited for the duration of the participation because of the potential to cause anaphylactic reactions in combination with the adsorber material.

### Provisions for post-trial care {30}

If any abnormalities are detected during the study examinations or if diseases are diagnosed, participants will be offered counseling sessions, as well as additional diagnostics and therapies, at our clinic. If IA is found to be an appropriate therapy for improving post-COVID symptoms, participants will receive guidance on how to continue the therapy or maintain the therapeutic effect of IA.

### Outcomes {12}

The primary outcome is the effect of the IA to the severity of the symptoms of a PCS as measured by five co-primary endpoints, i.e., the change of:

- Post-COVID functional scale (PCFS) with values from 0 to 4
- Chalder Fatigue Scale with values from 0 to 33
- Bell score with values from 0 to 100
- Multidimensional Fatigue Inventory (MFI-20) with values from 20 to 100 -Montreal cognitive Assessment (MoCA) ranging from 0-30
- Hand grip strength

before and 2 weeks after immunoadsorption and sham apheresis and the comparison of change of the median due to immunoadsorption with the change of the median due to sham apheresis. The various symptoms of PCS are difficult to capture due to their wide range. Therefore, several questionnaires have been selected to capture a broad spectrum of complaints and their potential improvement or worsening. Since the questionnaires, apart from the MOCA, assess symptom severity subjectively, the measurement of grip strength was chosen as an additional objective measure of potential improvement or worsening. We adjust for multiple testing by using the Bonferroni-Holm procedure.

The secondary outcome measures are as follows:

1. The number of treatment-emergent adverse events (TEAE), serious adverse events, and discontinuation of therapy because of adverse events and the comparison of them under immunoadsorption with the number of events under sham apheresis. Events are recorded for comparison 2 weeks after the end of each treatment cycle.
2. Prevalence of anti-adrenergic and anti-muscarinic autoantibodies in patients with PCS:
  - Proportion of subjects with evidence of anti-α1-adrenoreceptor antibodies (AB)
  - Proportion of subjects with evidence of anti-α2-adrenoreceptor AB
  - Proportion of subjects with evidence of anti-β1-adrenoreceptor AB
  - Proportion of subjects with evidence of anti-β2-adrenoreceptor AB
  - Proportion of subjects with evidence of anti-β3-adrenoreceptor AB
  - Proportion of subjects with detection of anti- M1 acetylcholine receptor AB
  - Proportion of subjects with detection of anti-M2 acetylcholine receptor AB
  - Proportion of subjects with detection of anti-M3 acetylcholine receptor AB
  - Proportion of subjects with detection of anti-M4 acetylcholine receptor AB

Concentration of autoantibodies will be measured at the time of the first examination before randomization for the first treatment modality.

3. Concentration of autoantibodies before and after IA and sham treatment (before therapy cycle 1/after therapy cycle 1 as well as before therapy cycle 2/after therapy cycle 2):
  - Concentration of anti-α1-adrenoreceptor antibodies (AB)
  - Concentration of anti-α2-adrenoreceptor AB
  - Concentration of anti-β1-adrenoreceptor AB
  - Concentration of anti-β2-adrenoreceptor AB
  - Concentration of anti-β3-adrenoreceptor AB
  - Concentration of anti- M1 acetylcholine receptor AB
  - Concentration of anti-M2 acetylcholine receptor AB
  - Concentration of anti-M3 acetylcholine receptor AB
  - Concentration of anti-M4 acetylcholine receptor AB

### Participant timeline {13}

**Fig. 1:**
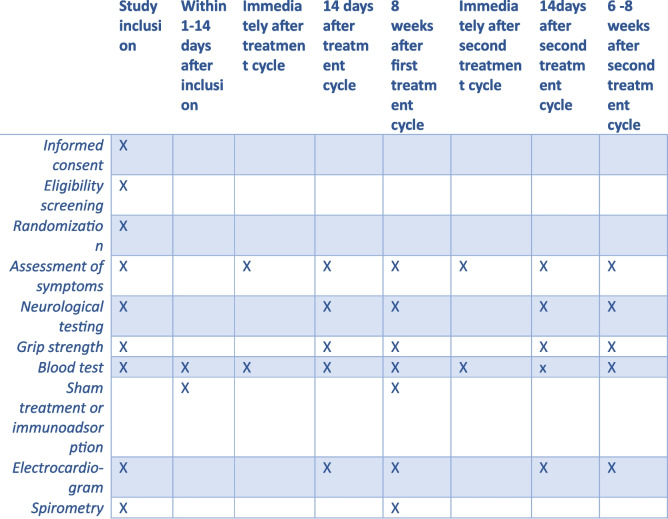
Time schedule

The participant timeline is shown in Fig. 1.

### Sample size {14}

Forty participants which meet the inclusion criteria will be recruited for this study. The primary outcome is the change of the severity of symptoms in particular the severity of cognitive impairment, which will be measured with the MoCA. In a published post-COVID cohort from Germany [18], the median MoCA score was 27 with an interquartile range of 25 to 28. The null hypothesis states that immunoadsorption does not improve the score. We aim to establish that immunoadsorption improves the MoCA score by at least 2 points, i.e., above the MoCA threshold, thus constituting a clinically relevant effect. As patients have the explicit option to withdraw after the first treatment sequence, there is some uncertainty whether a sufficient number of patients will actually take part in the second treatment. In this case, evaluation of the treatment would rely on a group comparison after the first sequence. Therefore, power calculation has been performed for a parallel group design. To achieve a power of 90%, a calculation using the G*Power software version 3.1.9.7 (Faul et al., Behav Res Methods 2009) indicates that two treatment groups with 19 participants each are required (effect size 1.0, one-sided Wilcoxon-Mann–Whitney test with an alpha level of 0.05, and 1:1 randomization). To account for potential dropout, the sample size is set at 2 × 20 participants, resulting in a total of 40 participants.

### Recruitment {15}

The participants will be recruited from the cohort of the Gutenberg Post-COVID Study (GLS), a cross-sectional observational study based in Mainz, Germany, including 700 people, with the objective to evaluate the prevalence of PCS in Rhineland-Palatine. All participants of the GLS with ongoing symptoms 6 months after a SARS-CoV-2 infection, where no other reasons for the complaints have been found in the context of the examinations during GLS visits, will be contacted via phone, email, or both and will be informed about the IAMPOCO trial. Alternatively, recruitment takes place through a post-COVID specialized medical practice, where patients are examined similarly to the GLS and alternative causes for the symptoms are ruled out. The exclusion of alternative explanations for the symptoms includes an echocardiography, a neurological assessment, and/or a cranial MRI, as well as a statement from a post-COVID specialty practice or clinic indicating that PCS is the most likely cause of the symptoms. If the persons are interested in participating in the IAMPOCO trial, an appointment for a personal meeting and briefing by the principal investigator or another member of the steering committee will be made, and the consent will be obtained.

## Assignment of interventions: allocation

### Sequence generation {16a}

Randomization as to whether the sham procedure or IA will be performed in the first treatment cycle, and thus consecutively which treatment will be performed in the second treatment cycle, will be performed using a random number generator.

### Concealment mechanism {16b}

The providers of the therapy are not blinded in this trial. For every participant, an electronic therapy protocol will be generated, which determines the therapy provided during the current therapy cycle. Participants are blinded of the current therapy mode by setting up the devices in a location spatially separated from the participant and then covering the device before, during, and after therapy.

### Implementation {16c}

Randomization is performed by the principal investigator or the deputy of the principal investigator at the time when a participant is enrolled into the study.

## Assignment of interventions: blinding

### Who will be blinded {17a}

The providers of the therapy are not blinded in this trial. Participants are blinded of the current therapy mode by setting up the devices in a location spatially separated from the participant and then covering the device before, during, and after Therapy.

### Procedure for unblinding if needed {17b}

As only participants are blinded of the sequence of therapy modalities, this is a single blinded study. Therefore, there is no apparent reason why participants should be unblinded except for the termination of their participation in the study.

## Data collection and management

### Plans for assessment and collection of outcomes {18a}

All data relevant for collecting outcomes, as well as safety parameters, have been predefined and will be collected from each study participant by the overseeing physicians at specified time points and entered anonymized into an electronic database.

The standardized trial database has been constructed using the Microsoft Excel software (Microsoft Corp., Berlin, Germany) in combination with the research electronic data capture (REDCap) hosted at University Medical Center of the Johannes Gutenberg-University Mainz. The database file will be uploaded to an internal storage that can be accessed only by the investigators.

### Plans to promote participant retention and complete follow-up {18b}

After providing their informed consent to participate in the study, patients receive a schedule that includes all necessary appointments for their study participation. Individual appointments can be rescheduled upon the patient’s request. One week prior to each appointment, participants will receive a reminder via email about the upcoming scheduled appointment.

### Data management {19}

The collected data is entered into the electronic database document by the researchers, which is password-protected and stored on an internal network drive. The document is regularly reviewed by the data manager for implausible or missing values. Any discrepancies are communicated to the researchers and resolved by them.

### Confidentiality {27}

An ID will be assigned to each study participant upon enrolment. All collected data will be associated solely with this ID. The principal investigator, data manager, and deputy of the principal investigator are the only individuals with access to a data file that links the IDs to the real names and birthdates of the participants. This file is password-protected and stored on an internal network drive, which is also password-protected.

### Plans for collection, laboratory evaluation, and storage of biological specimens for genetic or molecular analysis in this trial/future use {33}

Due to the high cost of laboratory analysis for specific autoantibodies and the fact that the kits used for analyzing 96 samples at once, serum samples from all participants are collected at different time points, pre-processed, and deep-frozen. Each sample is labeled with the participant ID and the time of collection. The analyses are conducted once 96 samples have been collected.

## Statistical methods

### Statistical methods for primary and secondary outcomes {20a}

Except for PCFS, the primary endpoints can be considered quantitative. Accordingly, mean, standard deviation, median, minimum, maximum, and quartiles will be determined, stratified by treatment (verum/placebo) and period (cycle 1/cycle 2). For the Chalder Fatigue Scale, the values from the visits 14 days after each treatment cycle will be used instead of the differences. A mixed linear model with treatment and period as fixed effects and patient as a random effect will be used to evaluate the significance of the influence of treatment and period on the changes under treatment. Marginal estimates and 95% confidence intervals for the effects of treatment and period will be reported, as well as *p* values for the main effects of treatment and period and their interactions. Additionally, it will be assessed whether a carry-over effect must be assumed. If the variables are clearly non-normally distributed, a Mann–Whitney test for crossover designs will be applied. For the ordinal variable PCFS, absolute and relative frequencies of each level and their changes (number of categories changed) under treatment will be determined. Furthermore, analogous to the mixed linear model to the quantitative primary endpoints, a generalized mixed linear model, which can be considered a generalization of an ordinal regression model, will be applied. Treatment and period will also be treated as fixed effects, with patient as a random effect.

Analogous to the primary endpoints, the differences between autoantibody levels after and before each treatment cycle will be determined to assess the effect of immunoadsorption on the anti-G-protein-coupled receptor autoantibodies as listed in the secondary outcomes section. The absolute and relative frequencies of patients with detectable antibodies will be reported. Additionally, mean, standard deviation, median, minimum, maximum, and quartiles will be determined, stratified by treatment (verum/placebo) and period (cycle 1/cycle 2). A mixed linear model with treatment and period as fixed effects and patient as a random effect will be used to evaluate the significance of the influence of treatment and period on the changes under treatment. Marginal estimates and 95% confidence intervals for the effects of treatment and period will be reported, as well as *p* values for the main effects of treatment and period and their interactions. It will also be assessed whether a carry-over effect must be assumed. If the variables are clearly non-normally distributed, a Mann–Whitney test for crossover designs will be applied.

Adverse events will be collected for up to 6 weeks after the end of each cycle. They will be listed stratified by period and treatment, and the highest severity grade will be reported. Furthermore, the absolute and relative frequencies of at least one adverse event occurring will be provided by period and treatment, thereby determining prevalence. The frequency of therapy discontinuation due to adverse events will also be reported, stratified by period and treatment.

### Interim analyses {21b}

There are no interim analyses planned.

### Methods for additional analyses (e.g., subgroup analyses) {20b}

Further analyses will be conducted to examine correlations between certain patient characteristics, such as the presence of specific autoantibodies, and the primary outcome. All parameters showing a correlation will then be included in a linear regression model for the primary outcome, such as improvement in fatigue symptoms measured by a reduction of 20 points in the MFI-20 score. The performance of the constructed linear regression model will be assessed based on the value of *R*^2^ and the significance of the coefficients to provide insights into the associations between specific characteristics and the clinical efficacy of the therapy.

### Methods in analysis to handle protocol non-adherence and any statistical methods to handle missing data {20c}

Applying the estimands concept, the primary strategy will be a so-called hypothetical strategy [19]. This approach includes all randomized patients along with the treatment cycles they participated in. That means patients who participated only in the first treatment cycle and subsequently withdrew from the study will remain in the analysis population. This corresponds to a hypothetical strategy and provides an estimate for the scenario in which all patients participate in the study as planned—assuming that dropouts are “missing at random.”

Multiple imputation is used to replace missing values in the primary and secondary endpoints. If patients withdraw from the study after the first treatment cycle, no values will be imputed for the second treatment cycle. Sensitivity analyses are planned to assess the robustness of the results of parameters with missing data. As a sensitivity analysis, a “principal stratum strategy” will be considered. In this approach, only patients who participated in both treatment cycles will be included in the analysis. This captures the stratum of the population that tolerates both treatments, regardless of the treatment sequence they were assigned to. Therefore, this constitutes a “complete case” analysis. In this case, the strategy corresponds to a classical per-protocol analysis.

If more than 10% of patients withdraw from the study after the first treatment sequence, we will also perform a comparison of results after the first treatment sequence between treatment groups.

### Plans to give access to the full protocol, participant-level data, and statistical code {31c}

The full protocol and statistical code will be accessible to the public on reasonable request. There is no plan of granting public access to the participant-level dataset. The results of the present trial will be presented at conferences and be published in a peer-reviewed journal to maximize the chances of dissemination of the results to the public. The results will be published, and a short form of the protocol is already published in the trial registry at clinicaltrials.gov.

## Oversight and monitoring

No committee is planned for this trial.

### Composition of the coordinating center and trial steering committee {5d}

The present trial is a single-center trial, so the University Medical Center Mainz is the coordinating and conducting center and the funding organization. All responsible persons are employees of the University Medical Center Mainz including the main investigator (MS), the principal investigator (JWM), the co-principal investigator (DK), and the responsible for statistics (AW, DK, MS, IS). Their roles primarily involve ensuring the smooth running of the study, reviewing the quality and completeness of data, ensuring patient safety, in addition to managing, analyzing, evaluating, writing the report, and its publication. No steering committee is formed.

### Composition of the data monitoring committee, its role and reporting structure {21a}

There is a data manager but no data monitoring committee has been formed.

### Adverse event reporting and harms {22}

SAEs (serious adverse events) are defined as unfavorable events that result in patient death, pose a life-threatening risk, lead to unexpected or prolonged hospitalization, or cause permanent or severe disability, irrespective of the plausibility of causal links with the trial interventions. Treating investigators will be responsible for managing all SAEs. The principal investigator, in consultation with the treating investigators, will assess the plausibility of the causal association. In the event of SAEs, the investigators will complete a predefined form and submit a report to the principal investigator. The principal investigator will collaborate with the other investigators to determine the trial’s continuation. Information regarding SAEs will be collected and recorded in the study database.

### Frequency and plans for auditing trial conduct {23}

No audits are planned.

### Plans for communicating important protocol amendments to relevant parties (e.g., trial participants, ethical committees) {25}

If there are any protocol amendments made, they will be communicated to all trial investigators and to all affected participants. The information at the trial registration will be updated additionally.

### Dissemination plans {31a}

Results of the present trial will be published in a peer-reviewed journal and will be presented on conferences to the public and to health professionals to gain the chances to improve the therapy of patients affected by PCS. Furthermore, the results will be released in the trial registry clinicaltrials.gov. Each participant will receive a report of their individual results after all participants have been treated and examined, and the data has been analyzed.

## Discussion

IAMPOCO was designed as a randomized controlled trial to evaluate the therapeutic effects of immunoadsorption in patients affected by PCS. Because of the growing number of patients with PCS and very little evidence regarding to treatment options, there was a need for a therapeutic trial. So far, there is only a small case series and individual case reports that describe partially conflicting results [14, 15]. Since some studies have demonstrated the presence of various autoantibodies in patients with PCS, a therapeutic effect of antibody-depleting therapy appears at least conceivable, providing evidence for the pathophysiological relevance of the autoantibodies [8, 9].

RCTs evaluating treatment methods are often conducted using a parallel-group design. However, in the planning of IAMPOCO, we opted for a crossover design, given that patients with PCS represent a highly heterogeneous population due to the lack of clear definitions for PCS and the difficulty in objectively assessing symptoms. The inclusion of a sham treatment arm was deemed necessary due to the anticipated placebo effect. For these reasons, comparing the effects of immunoadsorption and sham therapy within the same patients appeared more meaningful. Another potential strength of the IAMPOCO study could be the recruitment of participants from a cohort study on the prevalence of PCS or post-COVID specialized medical practice. This ensures the inclusion of patients who have been objectively evaluated by various disciplines and have largely ruled out alternative causes for their symptoms than PCS.

The search for suitable endpoints posed a significant challenge due to the difficulty or inability to objectively quantify many of the symptoms reported by patients, as well as the wide range of symptoms associated with PCS. The symptom burden and potential improvement are now being assessed using various scoring systems, a neurological test, and grip strength measurement to capture the entire spectrum of possible symptoms and their improvement as comprehensively as possible, while still aiming to objectively assess the symptoms. Secondly, it is not possible to blind the treating staff regarding the applied therapy method, as the structure and operation of the devices in the IA mode differ from that of the sham therapy. Currently, there are no data from randomized controlled trials regarding the therapy of PCS. Nevertheless, therapy methods such as IA are widely used, at least in Germany, without evidence of efficacy. Therefore, the results of the present RCT can make an important contribution to improving the therapy of PCS and enable evidence-based decision-making in treatment planning.

### Declaration of interests {28}

There are no competing interests for any of the authors.

### Data access {29}

The final study data are only accessible to the authors and the supporting statistician. It is planned for the analysis to be conducted by the authors. The study’s funders have access only to the results. Disclosure of contracts cannot take place as there are no contracts governing access rights to the study data.

### Dissemination policy: authorship {31b}

Authorship eligibility guidelines:

- Authors should have made substantial contributions to the conception and design of the study, acquisition of data, or analysis and interpretation of data.
- Authors should have actively participated in the drafting and critical revision of the article for important intellectual content.
- Authors should have reviewed and approved the final version of the article for publication.

Additionally, each author should meet all of the following criteria:

- Substantial contributions to the conception and design of the study or acquisition of data, or analysis and interpretation of data.
- Active involvement in the drafting of the article or revising it critically for important intellectual content.
- Final approval of the version to be published.
- Agreement to be accountable for all aspects of the work in ensuring that questions related to the accuracy or integrity of any part of the work are appropriately investigated and resolved.

Those who meet the criteria for authorship should be listed as authors. Contributors who do not meet all the authorship criteria should be acknowledged in the Acknowledgments section of the article. The use of professional writers is not intended.

### Informed consent materials {32}

A consent form has been submitted in a separated file.

## Trial registration

ClinicalTrials.gov NCT05841498. Registered on May 3, 2023. https://clinicaltrials.gov/study/NCT05841498

## Trial status

The trial is conducted with the protocol version 3.1, which was last updated February 27, 2023. The recruitment began in April 2023 and the enrolment commenced on May 8, 2023. Thirteen participants were enrolled until October 2023. The study protocol was submitted in January 2024 at this late stage because, due to the urgency of the research question and the numerous patient inquiries for treatment, we needed to prepare everything initially to commence the study before we could finalize the publication of the study protocol in the level of detail, as rightfully requested here. By June 2024, patient enrollment for the study was completed, and the study treatments and follow-up assessments continued until October 2024. At the time the study protocol was submitted for publication, only 13 patients had been enrolled in the study. Due to a review process lasting over 9 months, the study treatment was completed by the time the protocol was published. In August 2024, the statistical methods were revised, and the statistical analysis plan was developed, resulting in protocol version 3.3.

## Supplementary Information

Supplementary Material 1.

Supplementary Material 2.

Supplementary Material 3.

## Data Availability

Since the submitted manuscript is a study protocol and the study is currently ongoing, there are no data available regarding the study results at this time. The provided manuscript does not present any results either.
