# Supplementary material for: Immunoadsorption study Mainz in adults with post-COVID syndrome (IAMPOCO)—a single-blinded sham-controlled crossover trial to evaluate the effect of immunoadsorption on post-COVID syndrome
Source: Trials. 2025 Apr 3;26:119. doi: 10.1186/s13063-025-08825-7 (PMC11966871; doi:10.1186/s13063-025-08825-7)
Supplement: Supplementary file 2 — Supplementary Material 2. [file 13063_2025_8825_MOESM2_ESM.pdf]

UNIVERSITÄTSmedizin. MAINZ

I. Medizinische Klinik und Poliklinik

Direktor:
Univ.-Prof. Dr. med. Peter R. Galle

Prof Dr. med. Julia Weinmann-Menke
Leiterin der Schwerpunks Neprhologie
Schwerpunkt Nephrologie, Rheumatologie und klinische Immunologie
Geb. 605, EG
Langenbeckstr. 1
55131 Mainz
Telefon:+49 (0) 6131 17-7128
Telefax:+49 (0) 6131 17-3492

Einwilligungserklärung für PatientInnen

„ImmunAdsorptionstudie Mainz bei Erwachsenen mit Post-COVID (IAMPOCO)“

wissenschaftliche Studie zur:

Prüfung des Effekts einer Immunadsorptionstherapie auf die Symptomlast von Erwachsenen mit Post-COVID-Syndrom.

Namen der Patienten in Druckbuchstaben: ____

- Ich bin von Herrn / Frau _________________________ über Wesen, Bedeutung und Tragweite der Studie sowie die sich für mich daraus ergebenden Anforderungen aufgeklärt worden. Ich habe darüber hinaus den Text der Patientenaufklärung und dieser Einwilligungserklärung gelesen.
- Ich hatte ausreichend Zeit, Fragen zu stellen und mich zu entscheiden. Aufgetretene Fragen wurden mir vom Studienarzt beantwortet.
- Ich weiß, dass ich meine freiwillige Mitwirkung jederzeit beenden kann, ohne dass mir daraus Nachteile entstehen.

Ich erkläre mich bereit, an der Studie teilzunehmen.

1. Ich willige ein, dass personenbezogene Daten über mich, wie in der Informationsschrift beschrieben erhoben und in Papierform sowie auf elektronischen Datenträgern in der I. Medizinischen Klinik der Universitätsmedizin Mainz aufgezeichnet werden. Zu diesem Zweck entbinde ich die mich behandelnden Ärzte von der ärztlichen Schweigepflicht.

Soweit erforderlich, dürfen die erhobenen Daten pseudonymisiert (verschlüsselt) weitergegeben werden:

a) an **Herr PD Dr. Arndt Weinmann (Oberarzt der I. Medizinischen Klinik)** zum Zweck der wissenschaftlichen Auswertung,

b) im Falle unerwünschter Ereignisse: an die jeweils zuständige Ethik-Kommission und zuständige Behörden sowie von dieser an die Europäische Datenbank.

2. Ich bin darüber aufgeklärt worden, dass ich meine Einwilligung jederzeit widerrufen kann. Im Falle des Widerrufs werden keine weiteren Daten mehr erhoben. Ich kann in diesem Fall die Löschung der Daten verlangen

3. Ich willige ein, dass die Daten nach Beendigung oder Abbruch der klinischen Prüfung mindestens 10 Jahre aufbewahrt werden.

Kontaktdaten des behandelnden Arztes:

____

(Name und Vorname in Druckschrift)

____

(Adresse)

____

(Telefon/Fax)

Ich willige in die Verarbeitung der genannten Daten ein.

Ein Exemplar der Informationsschrift und der Einwilligungserklärung habe ich erhalten. Ein Exemplar verbleibt im Prüfzentrum.

Unterschrift des Teilnehmers/der Teilnehmerin

____

Name des Patienten in Druckbuchstaben

______________ ______________________________

Datum/Ort Unterschrift des Patienten

Erklärung und Unterschrift des aufklärenden Arztes/der aufklärenden Ärztin

Ich habe das Aufklärungsgespräch geführt und die Einwilligung des Patienten eingeholt.

____

Name des Prüfarztes/der Prüfärztin in Druckbuchstaben

______________ ______________________________

Datum Unterschrift des aufklärenden Prüfarztes/der Prüfärztin
