# Supplementary material for: Immunoadsorption study Mainz in adults with post-COVID syndrome (IAMPOCO)—a single-blinded sham-controlled crossover trial to evaluate the effect of immunoadsorption on post-COVID syndrome
Source: Trials. 2025 Apr 3;26:119. doi: 10.1186/s13063-025-08825-7 (PMC11966871; doi:10.1186/s13063-025-08825-7)
Supplement: Supplementary file 3 — Supplementary Material 3. [file 13063_2025_8825_MOESM3_ESM.pdf]

UNIVERSITÄTSmedizin. MAINZ

I. Medizinische Klinik und Poliklinik

Direktor:
Univ.-Prof. Dr. med. Peter R. Galle

Prof. Dr. med. Julia Weinmann-Menke
Schwerpunkt Nephrologie, Rheumatologie und klinische Immunologie
Geb. 605, EG
Langenbeckstr. 1
55131 Mainz
Telefon:+49 (0) 6131 17-6774
Telefax:+49 (0) 6131 17-3492

Version 3.1

**Patienteninformation für PatientInnen**

**„Immunadsorptionsstudie Mainz bei Erwachsenen mit Post-COVID-Syndrom zur Überprüfung der therapeutischen Wirkung auf die Symptomlast“**

Sehr geehrte Patient*Innen,

wir möchten Sie fragen, ob Sie an einer wissenschaftlichen Studie teilnehmen möchten. Sie hatten mindestens einen positiven SARS-COV-2-Nachweis mittels PCR, sowie neue oder aus der SARS-COV-2-Infektion andauernde Symptome 3 Monate nach positivem PCR-Test, die über mindestens 2 Monate andauern. Durch diese Studie soll die Wirksamkeit der Behandlung mit einer extrakorporalen Immunadsorption untersucht werden. Hierbei handelt es sich um ein Verfahren, bei dem Blut über einen Venenzugang ausgeleitet wird (ca. 50ml/min) und durch eine Art Filter geleitet wird, wobei Antikörper und andere Botenstoffe des Immunsystems im Filter zurückgehalten werden. Das so aufgereinigte Blut wird dann wieder über einen weiteren venösen Zugang zurück in den Körper geleitet.

Für die Studienteilnahme sind ambulante Evaluationsuntersuchungen, die ca. einen Tag Zeit in Anspruch nehmen, notwendig und im Anschluss zwei stationäre Aufenthalte über je 7 Tage. Da Sie bereits an Gutenberg Long-COVID-Studie teilgenommen haben, können wir viele Untersuchungsergebnisse aus dieser Studie übernehmen.

Verantwortliche Studienleiterin ist Frau Professor Dr. Julia Weinmann-Menke, Leiterin des Schwerpunkts Nephrologie der Universitätsmedizin Mainz. Insgesamt sollen 40 Patient*innen an der Studie teilnehmen, welche alle in der I. Medizinischen Klinik der Universitätsmedizin Mainz betreut werden. Ihre Teilnahme an der Studie hat keinen Einfluss auf eine anderweitige medizinische Behandlung in der Universitätsmedizin Mainz.

Die Teilnahme an dieser Studie ist freiwillig. Sie werden nur dann einbezogen, wenn Sie dazu schriftlich Ihre Einwilligung erklären. Sofern Sie nicht an der Studie teilnehmen oder später aus ihr ausscheiden möchten, entstehen Ihnen dadurch keine Nachteile. Sie können jederzeit, auch ohne Angabe von Gründen, Ihre Einwilligung mündlich oder schriftlich widerrufen.

Die Studie wurde der zuständigen Ethikkommission vorgelegt. Sie hat keine Einwände erhoben.

**Worum handelt es sich?**

Da Sie noch mindestens 3 Monate nach einer Infektion mit dem SARS-COV-2-Virus unter Symptomen, die während der akuten Infektion begonnen haben oder sich nach abheilen der akuten Infektion entwickelt haben und diese nun schon mindestens 2 Monate lang andauern, leiden Sie wahrscheinlich unter dem sogenannten Post-COVID-Syndrom. Hierbei handelt es sich um einen Symptomkomplex, der nach einer Infektion mit dem SARS-COV-2-Virus auftreten kann. Die genaue Ursache dieses Syndroms ist bislang nicht verstanden. Es sind ähnliche Krankheitsbilder nach anderen Viruserkrankungen bekannt, beispielsweise nach einer Infektion mit dem Epstein-Barr-Virus, dem Erreger des Pfeiffer'schen Drüsenfiebers. Als Ursachen für derartige Krankheitsbilder kommen unter anderem Mechanismen in Frage, bei denen sich das Immunsystem gegen den eigenen Körper richtet, da bestimmte Eiweiße an der Oberfläche der Körperzellen Ähnlichkeit mit Eiweißen der jeweiligen Virushülle besitzen, was als Autoimmunität bezeichnet wird. Auch eine Überstimulation des Immunsystems durch die Virusinfektion kann als Ursache in Betracht gezogen werden.

Wie lange die Symptome im Rahmen des Post-COVID-Syndroms andauern und ob sie spontan wieder verschwinden ist ebenfalls noch nicht hinreichend geklärt. Ebenso ist derzeit nicht klar, welche Therapien die Symptome lindern können oder auch komplett verschwinden lassen können.

Bei einigen Autoimmunerkrankungen spielt die Immunadsorption in der Therapie eine Rolle und zeigt auch Therapieerfolge. Diese Therapieerfolge beruhen darauf, dass durch die Immunadsorption wirksam Antikörper aus dem Blut „gefiltert" werden, strenggenommen adsorbiert werden, hierunter auch der Teil der Antikörper, der sich gegen Strukturen des eigenen Körpers richtet und somit zur Autoimmunerkrankung führt.

Da es Hinweise darauf gibt, dass Autoimmunmechanismen auch beim Post-COVID-Syndrom eine Rolle spielen könnten, wollen wir im Rahmen dieser Studie untersuchen, ob die Immunadsorption hilft, Symptome eines Post-COVID-Syndroms zu lindern oder dieses sogar vollständig verschwinden. Auch wollen wir im Rahmen der mit der Studienteilnahme verbundenen Laboruntersuchungen nach Hinweisen auf eine autoimmune Genese des Post-COVID-Syndroms suchen um die Ursache besser erklären zu können.

**Verlauf und Dauer der Studie**

Wenn Sie sich bereit erklären, an der Studie teilzunehmen, werden zunächst einige vorausgehende Untersuchungen bei Ihnen durchgeführt. Diese beinhalten verschiedene Fragebögen und psychologische Tests, neurologische Tests mittels Fragebögen und kleineren Aufgaben, EKG, Lungenfunktionsuntersuchung, Kraftmessung, Prüfung der Sensibilität, Urinanalysen sowie Blutentnahmen. Sollten bei diesen Untersuchungen keine anderen Ursachen als Erklärung für Ihre Symptome gefunden

werden, finden bei Ihnen Immunadsorptionsbehandlungen statt.
Da wir mittels diese Studie den Effekt der Immunadsorption auf Ihre Symptome prüfen wollen, ist es wichtig weitere Einflussfaktoren auf die Schwere Ihrer Symptome möglichst auszuschließen. Dies geschieht dadurch, dass außer den 5 Immunadsorptionen auch 5 Scheinbehandlungen stattfinden, bei denen Sie an die gleiche Maschine wie zur Immunadsorption angeschlossen werden, das Blut jedoch ohne den entsprechenden Filter, der die Antikörper aus dem Blut entfernt, in Ihre Venen zurückgeleitet wird. Zum Zeitpunkt der Therapie sind die Maschinen mittels eines Vorhangs für Sie verdeckt, sodass Sie nicht wissen, ob Sie Immunadsorption erhalten oder die Scheinbehandlung. Ob bei ihnen zunächst 5 Immunadsorptionen oder zuerst 5 Scheinbehandlungen stattfinden, wird per Los entschieden. Auch erfahren Sie erst nach Abschluss der Studie, ob Sie zuerst Scheinbehandlungen oder Immunadsorptionen hatten. Nach dem ersten Therapiezyklus (5 Immunadsorptionen oder 5 Scheinbehandlungen) erfolgt dann eine Therapiepause von 8 Wochen, um die möglichen Effekte der vorangegangenen Therapie auf den nächsten Zyklus weitgehend ausschließen zu können. Vor und nach dieser Therapiepause können Sie gemeinsam mit einem der Studienärzte entscheiden, ob Sie sich auch dem zweiten Therapiezyklus unterziehen wollen. Vor und nach jedem Therapiezyklus erfolgen wieder Untersuchungen mittels verschiedener Fragebögen und psychologischer Tests, neurologischer Tests mittels Fragebögen und kleineren Aufgaben, EKG, Lungenfunktionsuntersuchung, Kraftmessung, Prüfung der Sensibilität, Urinanalysen sowie Blutentnahmen. Für die 5 Immunadsorptionen ebenso wie für die 5 Scheinbehandlungen ist jeweils ein 9-tägiger stationärer Aufenthalt in der Universitätsmedizin notwendig.
6 Wochen nach dem letzten Therapiezyklus erfolgt eine ambulante Vorstellung mit erneuten neurologischen und psychologischen Tests, EKG, Lungenfunktionsuntersuchung und Blutentnahme.

**Ablauf der Immunadsorption**
Zunächst legt der Arzt Ihnen in jeden Arm eine Nadel in ein Blutgefäß (Vene). Es kann für die Behandlung erforderlich sein, dass alternativ bzw. zusätzlich ein Kunststoffschlauch (Katheter) in ein größeres Blutgefäß am Hals gelegt wird. Wie beim Blutspenden wird das Blut an einem Armzugang zur Behandlung über ein Schlauchsystem entnommen. Damit das Blut nicht gerinnt, wird ein blutverdünnendes Mittel (z.B. Heparin oder Zitrat) zugeführt.
Das Plasma (flüssige Blutbestandteile ohne Zellen) wird mit einem ersten Filter aus dem Blut abgetrennt und dann zu einem zweiten speziellen Filter (Adsorber) geleitet. Verschiedene Adsorbermaterialien (eventuell auch in Kombination) binden die Antikörper und Immunkomplexe und entfernen sie somit aus dem Plasma. Das gereinigte Plasma wird Ihnen anschließend zusammen mit den Blutzellen über den zweiten Armzugang wieder zugeführt.

**Was wird von Ihnen bei einer Teilnahme erwartet?**
Im Rahmen der Studienteilnahme werden bei Ihnen zu Beginn der Studienteilnahme sowie vor und nach jedem Behandlungszyklus verschiedene neurologische und psychologische Untersuchungen durchgeführt, meist mittels Fragebögen und Testaufgaben. Weiterhin erfolgt eine Lungenfunktionsuntersuchung, ein EKG, sowie die Erhebung der Vitalparameter (dies erfolgt mehrmals und auch kontinuierlich während der extrakorporalen Therapie).

Bei Studieneinschluss ebenso wie vor und nach jedem Therapiezyklus sind Blutentnahmen von ca. 70ml Blut notwendig. Während der extrakorporalen Therapie wird Blut am Gerät entnommen um die Blutsalze und den pH-Wert des Blutes zu überwachen.
Um einen reibungslosen Ablauf gewährleisten zu können und zur Erhöhung Ihrer Sicherheit ist für jeden der beiden Therapiezyklen ein 7-tägiger stationärer Aufenthalt notwendig.

**Mögliche Risiken und Nebenwirkungen:**
Die Immunadsorption ist ein sehr sicheres und risikoarmes Routineverfahren. Trotz aller Sorgfalt kann es jedoch zu – u.U. auch lebensbedrohlichen – Komplikationen kommen, die weitere Behandlungsmaßnahmen/Operationen erfordern. Die Häufigkeitsangaben sind eine allgemeine Einschätzung und sollen helfen, die Risiken untereinander zu gewichten. Sie entsprechen nicht den Definitionen für Nebenwirkungen in den Beipackzetteln von Medikamenten. Vor- und Begleiterkrankungen sowie individuelle Besonderheiten können die Häufigkeiten von Komplikationen wesentlich beeinflussen.

- Bei Einnahme von sogenannten ACE-Hemmern, einer bestimmten Klasse von Blutdrucksenkenden Medikamenten besteht das Risiko von schweren Blutdruckabfällen bei Behandlungen mit tryptophanhaltigen Adsorbern wie sie im Rahmen der Studie verwendet werden. Vor Studienbeginn gehen wir gemeinsam mit Ihnen ihre Medikamente durch und prüfen, ob sie diese Substanzen einnehmen. Um das Risiko weiter zu minimieren **muss jede Änderung Ihrer Medikation einem der Studienärzte unverzüglich mitgeteilt werden.**
- Bei der Behandlung können **milde vorübergehende Nebenwirkungen**, die keiner speziellen Behandlung bedürfen, auftreten. Dies sind z.B. Kältegefühl, Husten, Benommenheit, Blutdruckschwankungen, Kopfschmerzen, Tränenfluss, Herzrasen, Juckreiz, Schnupfen, Gefühlsstörung, Tinnitus (Ohrgeräusch) oder Erbrechen (bei ca.10% der Behandlungen).
- Es kann zu niedrigem oder zu erhöhtem Blutdruck kommen. Dies äußert sich u.U. in **Kopfschmerzen**, **Kreislaufreaktionen** wie **Schwindel, kaltem Schweiß, Luftnot** oder **Übelkeit**, selten auch in **Bewusstlosigkeit** (Blutdruckschwankungen bei ca. 20% der Behandlungen).
- Sehr selten treten **Schmerzen im Brustkorb** und **Rücken** auf. Sie können u.a. Anzeichen von Durchblutungsstörungen der Herzkranzgefäße (Angina pectoris, Herzinfarkt) oder einer Lungenembolie sein. Oft besteht jedoch kein direkter Zusammenhang mit der Behandlung. In der Regel wird in einem solchen Fall eine weitere Diagnostik und eventuell eine spezielle Therapie eingeleitet (bei ca. 4% der Behandlungen).
- **Entzündungen an den Armzugängen** und **Fehllage von Nadeln oder Kathetern** können selten **Schmerzen**, insbesondere am Gefäßzugang, oder **Blutergüsse** (Hämatome) und **Blutungen** verursachen (bei ca. 5% der Behandlungen). Sie bilden sich in der Regel von selbst zurück. Ggf. können sie medikamentös behandelt werden.
- Wegen der erforderlichen medikamentösen Blutverdünnung können auch **Blutungen in das Gehirn** (z.B. chronisches subdurales Hämatom), in den **Magen-Darm-Bereich** und in den **Herzbeutel** entstehen. Die Blutungsgefahr ist erhöht, wenn Sie bereits gerinnungshemmende Medikamente (z.B. Marcumar®, Aspirin®, Heparin) einnehmen. Das Risiko für Blutungen und Hämatome beträgt insgesamt ca. 9%, webei es sich

meist um leichte Blutungen handelt. Lebensbedrohliche Blutungen, die eine operative Blutstillung und/oder Bluttransfusion erfordern, sind aber selten. Kommt eine Fremdbluttransfusion in Betracht, werden Sie über die Durchführung und Risiken (z.B. Infektionen, u.U. auch mit unbekannten Krankheitserregern) gesondert aufgeklärt. Das Risiko einer HIV-Infektion oder Hepatitis ist dabei äußerst gering.

- Wenn die Behandlung mit einem Katheter durchgeführt wird, kann es zur Blutgerinnung, u.U. mit Ausbildung eines **Gefäßverschlusses des Blutgefäßes (Thrombose)**, in die der Katheter eingeführt wurde, kommen (in ca. 4% der Fälle). Eventuell wird dann mit einem gerinnungshemmenden Medikament behandelt.
- Die Anwendung von blutverdünnenden Stoffen (z.B. Heparin) erhöht die Blutungsneigung und kann zu einer schweren – im Falle von Heparin u.U. lebensbedrohenden – **Störung der Blutgerinnung** führen (Thrombozytopenie, HIT II) (kann 0,1-5% der behandelten Patient*innen betreffen).
- Häufig treten **Veränderungen der Blutsalze** wie Kalzium, Kalium oder Magnesium auf. Mögliche Folgen sind **Muskelkrämpfe**, insbesondere in den Waden, die in der Regel durch relativ einfache ärztliche Maßnahmen (z.B. Tabletten oder Kochsalzinfusion) behoben werden können (betrifft ca. 5-8% der durchgeführten Behandlungen). Weiter kann es zu **Herzrhythmusstörungen** kommen, vor allem bei vorgeschädigtem Herzen und unter Medikamenteneinnahme (z.B. Digitalis). Sehr selten nehmen diese Rhythmusstörungen lebensbedrohliche Formen bis hin zum **Kreislaufstillstand** an.
- Dringt trotz aller Sorgfalt Luft in das Schlauchsystem ein, kann sie über den Kreislauf bis zur Lunge gelangen und ein Blutgefäß verschließen (**Luftembolie**), dies kommt allerdings in weniger als 1% der Fälle vor. In Einzelfällen kann durch eine Luftembolie, ebenso wie durch ein in den Blutkreislauf gelangtes Blutgerinnsel, u.a. ein **Schlaganfall** mit Lähmungen oder Sehverlust sowie ein **Herz- oder Atemstillstand** verursacht werden. Mögliche Anzeichen sind Schmerzen im Brustkorb, Husten, Atemnot, Blaufärbung der Haut und/ oder der Schleimhäute (Zyanose), Kopfschmerzen, Schwindel, Ohrgeräusch, Sehstörungen bis hin zu Krampfanfall sowie Bewusstlosigkeit.
- **Infektionen**, u.U. mit Fieber, die meist antibiotisch gut behandelbar sind, können auftreten. Eine Keimausbreitung in die Blutbahn bis hin zur lebensbedrohlichen Blutvergiftung (**Sepsis**) oder Herzklappenentzündung (**Endokarditis**) oder andere schwerwiegende Infektionen, die eine intensivmedizinische Behandlung erfordern, sind selten (keine Literaturangaben zur Häufigkeit verfügbar).
- Aus dem Blutplasma werden nicht nur die krankheitsverursachenden Antikörper **herausgefiltert**, sondern teilweise auch **gesunde Antikörper**, die Infekte abwehren. Damit **erhöht sich die Infektanfälligkeit**. Daher erhalten Sie u.U. nach der Behandlung Immunglobuline (Immunprophylaxe) (In der Literatur ist die Häufigkeit von Infekten nach Immunadsorption nicht höher als nach Scheinbehandlungen angegeben).
- **Allergie/Unverträglichkeit** (Häufigkeit ca. 3%) (z.B. auf Begleitstoffe des Schlauch- oder Filtermaterials, Medikamente, Desinfektionsmittel, Latex) kann zu einem akuten Kreislaufschock führen, der intensivmedizinische Maßnahmen erfordert. Äußerst selten sind schwerwiegende, u.U. bleibende Schäden (z.B. Organversagen, Hirnschädigung, Lähmungen).
- **Haut-/Gewebe-/Nervenschäden** durch die Lagerung und eingriffsbegleitende Maßnahmen (z.B. Einspritzungen, Desinfektionen) sind selten. Mögliche, u.U. dauerhafte Folgen: Schmerzen, Entzündungen, Absterben von Gewebe, Narben sowie Empfindungs-, Funktionsstörungen, Lähmungen (z.B. der Gliedmaßen) (Häufigkeit <1%).

**Mögliche Nutzen aus Ihrer Teilnahme an dieser Forschungsstudie:**

Sollte die Immunadsorption gegen Ihre Beschwerden, die durch das Post-COVID-Syndrom verursacht sind wirken, haben Sie durch die Studienteilnahme den Nutzen, dass es Ihnen nach der Teilnahme zumindest eine Zeit lang bessergehen wird. Kann mit der Studie ein Nutzen der Immunadsorption gezeigt werden, kann auch ein Antrag bei Ihrer Krankenkasse gestellt werden, damit die Kosten für eine längerfristige Therapie mittels Immunadsorption übernommen werden. Weiterhin erhoffen wir uns von der Studie auch neue Erkenntnisse zur Entstehung der Symptome eines Post-COVID-Syndroms, welche Ihnen als Betroffenem auch zu Gute kommen können, wenn sich hieraus weitere mögliche Therapieansätze ergeben.

Die während Ihrer Studienteilnahme durchgeführten und erfassten Untersuchungsergebnisse werden Ihnen nach Abschluss Ihrer Studienteilnahme mitgeteilt. Sollten bislang nicht festgestellte Krankheiten bei Ihnen gefunden werden, werden Sie hierüber unverzüglich informiert und zu Behandlungsmöglichkeiten beraten.

**Patientenversicherung**

Damit Sie im Falle von möglichen unerwünschten Wirkungen oder bleibenden Schäden bis zu einer Schadenssumme von 500 000€, die aus der Studienteilnahme resultieren, abgesichert sind, wurde eine Patientenversicherung bei der HDI-Versicherungsgesellschaft abgeschlossen.

**Datenschutz**

- Rechtsgrundlage für die Datenverarbeitung ist Ihre freiwillige Einwilligung (Art. 6 Abs. 1 Buchst. c) DSGVO).
- Der Verantwortliche für die Datenverarbeitung ist: Die I. Medizinische Klinik der Universitätsmedizin Mainz

Alle Angaben und Daten werden selbstverständlich nach den gesetzlichen Vorgaben des Bundesdatenschutzgesetztes erhoben. Die Blutproben, Urinproben und persönlichen Daten der Studienteilnehmer werden noch im Untersuchungsraum pseudonymisiert (d.h. kodiert ohne Angaben von Name, Anschrift, Initialen oder Ähnliches) und können dann nur noch durch einen Entschlüsselungscode Ihrer Person zugeordnet werden. Dieser Code ist nur dem Studienleiter bekannt. Für die Studie selbst werden Ihre sogenannten Identifikationsdaten (Name, Vorname, Geburtsdatum) erfasst. Jedem registrierten Patienten wird dann ein Pseudonym (Zahlencode) für die Erfassung der medizinischen Daten zugeordnet. Die Übermittlung Ihrer Identifikationsdaten ist erforderlich, um alle nacheinander anfallenden medizinischen Untersuchungsdaten richtig zuordnen zu können, auch wenn verschiedene Ärzte an Ihrer Behandlung beteiligt sind. Alle für die Studie wichtigen medizinischen Daten zu Ihrem Krankheitsverlauf werden dann ohne Namensbezug in pseudonymisierter Form für 10 Jahre gespeichert. Die Auswertungen der Daten erfolgen ausschließlich pseudonymisiert. Falls es zu einer Veröffentlichung der Ergebnisse kommt (z.B. auf Fachtagungen oder in Fachzeitschriften), erfolgt dieses ausschließlich anonym, so dass ein Rückschluss auf eine einzelne Person nicht möglich ist.

**Sind mit der Datenverarbeitung Risiken verbunden?**

Bei jeder Erhebung, Speicherung, Nutzung und Übermittlung von Daten bestehen Vertraulichkeitsrisiken (z.B. die Möglichkeit, die betreffende Person zu identifizieren). Diese Risiken lassen sich nicht völlig ausschließen und steigen, je mehr Daten miteinander verknüpft werden können. Der Initiator der Studie versichert Ihnen, alles nach dem Stand der Technik Mögliche zum Schutz Ihrer Privatsphäre zu tun und Daten nur an Stellen weiterzugeben, die ein geeignetes Datenschutzkonzept vorweisen können. Medizinische Risiken sind mit der Datenverarbeitung nicht verbunden.

**Kann ich meine Einwilligung widerrufen?**

Sie können Ihre jeweilige Einwilligung jederzeit ohne Angabe von Gründen schriftlich oder mündlich widerrufen, ohne dass Ihnen daraus ein Nachteil entsteht. Wenn Sie Ihre Einwilligung widerrufen, werden keine weiteren Daten mehr erhoben. Die bis zum Widerruf erfolgte Datenverarbeitung bleibt jedoch rechtmäßig.

Sie können im Fall des Widerrufs auch die Löschung Ihrer Daten verlangen.

**Welche weiteren Rechte habe ich bezogen auf den Datenschutz?**

Sie haben das Recht, vom Verantwortlichen Auskunft über die von Ihnen gespeicherten personenbezogenen Daten (einschließlich der kostenlosen Überlassung einer Kopie der Daten) zu verlangen. Ebenfalls können Sie die Berichtigung unzutreffender Daten sowie gegebenenfalls eine Übertragung der von Ihnen zur Verfügung gestellten Daten und die Einschränkung ihrer Verarbeitung verlangen.

Bitte wenden Sie sich im Regelfall an das Studienzentrum, denn allein das Studienzentrum kann aufgrund des Pseudonymisierungsprozesses vollumfänglich auf Ihre Daten zugreifen bzw. entsprechende Auskünfte geben. Der Initiator der Studie kann vor diesem Hintergrund nur sehr begrenzt helfen.

Bei Anliegen zur Datenverarbeitung und zur Einhaltung der datenschutzrechtlichen Anforderungen können Sie sich auch an folgende Datenschutzbeauftragten der Universitätsmedizin Mainz wenden:

**datenschutz@unimedizin-mainz.de**

Sie haben ein Beschwerderecht bei jeder Aufsichtsbehörde für den Datenschutz. Eine Liste der Aufsichtsbehörden in Deutschland finden Sie unter

https://www.bfdi.bund.de/DE/Infothek/Anschriften_Links/anschriften_links-node.html

**Ansprechpartner für Fragen zur Studie**

1. Frau Professor Weinmann-Menke
   Julia.Weinmann-Menke@unimedizin-mainz.de
   Tel: 06131-176774

2. Herr Dr. Marco Stortz
   Marco.stortz@unimedizin-mainz.de
   Tel: 06131- 172213

Vorsorglich werden Sie darauf hingewiesen, dass eine Versicherung für nicht schuldhaft verursachte Schäden, die im Zusammenhang mit der Studie auftreten können, abgeschlossen wurde. Ein Versicherungsschutz besteht damit nicht nur, wenn den Arzt oder einen anderen Mitarbeiter der Studienstelle der Vorwurf eines schuldhaften Fehlverhaltens trifft. Zugunsten des Studienteilnehmers können dabei in bestimmten Fällen Beweiserleichterungen eintreten (Bürgerliches Gesetzbuch, §630h: zur Beweislast bei Haftung für Aufklärungs- und Behandlungsfehler): Wegeunfälle sind ebenfalls versichert. Für die studienbedingten Blutentnahmen besteht Versicherungsschutz über die Unfallkasse des Landes Rheinland-Pfalz (Orensteinstr.10, 56626 Andernach, Tel 02632 960-0, Fax 02632 960-1000).

Wenn Sie sich zur Teilnahme entschließen, bitten wir Sie, Ihre Einwilligung durch Ihre Unterschrift zu erklären. Die Einwilligungserklärung muss von Ihnen persönlich datiert und unterschrieben werden. Von der Einwilligungserklärung und von dieser Patienteninformation wird Ihnen eine Kopie ausgehändigt.

Falls Sie weitere Fragen bezüglich der Studie haben, stehen wir Ihnen gerne zur Verfügung.

Mit freundlichen Grüßen

Frau Prof. Dr. med. Julia Weinmann-Menke
Leiterin des Schwerpunktes Nephrologie
I. Medizinische Klinik und Poliklinik, Schwerpunkt Rheumatologie und Nephrologie
Universitätsmedizin Mainz
Email: Julia.Weinmann-Menke@unimedizin-mainz.de

Dr. med. Marco Stortz
Assistenzarzt
I. Medizinische Klinik und Poliklinik, Schwerpunkt Rheumatologie und Nephrologie
Universitätsmedizin Mainz
Email: marco.stortz@unimedizin-mainz.de
